# Cluster Differences in Antibiotic Resistance, Biofilm Formation, Mobility, and Virulence of Clinical *Enterobacter cloacae* Complex

**DOI:** 10.3389/fmicb.2022.814831

**Published:** 2022-04-06

**Authors:** Shixing Liu, Liqiong Chen, Lingbo Wang, Beibei Zhou, Dandan Ye, Xiangkuo Zheng, Yishuai Lin, Weiliang Zeng, Tieli Zhou, Jianzhong Ye

**Affiliations:** ^1^Key Laboratory of Clinical Laboratory Diagnosis and Translational Research of Zhejiang Province, Department of Clinical Laboratory, The First Affiliated Hospital of Wenzhou Medical University, Wenzhou, China; ^2^School of Laboratory Medicine and Life Science, Wenzhou Medical University, Wenzhou, China

**Keywords:** *Enterobacter cloacae* complex, clinical distribution, resistance, biofilm, virulence

## Abstract

Due to the lack of research on the characteristics of different clusters of *Enterobacter cloacae* complex (ECC), this study aimed to characterize and explore the differences among species of the ECC. An analysis based on *hsp60* showed that *Enterobacter hormaechei* was predominant in ECC. Interestingly, the antibiotic resistance rates of clusters were different, among which *E. hormaechei* subsp. *steigerwaltii* (cluster VIII) and *Enterobacter cloacae* IX (cluster IX) possessed high resistant rates to ciprofloxacin and levofloxacin, but cluster II (*Enterobacter kobei*) had low resistant rates. Cluster II exhibited a strong biofilm formation ability. Different motility and protease production ability were shown for distinct clusters. A PCR analysis showed that clusters I, III, VI, VIII, and IX carried more virulence genes, while cluster II had fewer. Clusters I, VIII, and IX with high pathogenicity were evaluated using the *Galleria mellonella* infection model. Thus, the characteristics of resistance, biofilm-forming ability, mobility, and virulence differed among the clusters. The strains were divided into 12 subgroups based on *hsp60*. The main clusters of ECC clinical strains were I, II, III, VI, VIII, and IX, among which IX, VIII, and I were predominant with high resistance and pathogenicity, and cluster II (*E. kobei*) was a special taxon with a strong biofilm formation ability under nutrient deficiency, but was associated with low resistance, virulence, and pathogenicity. Hence, clinical classification methods to identify ECC subgroups are an urgent requirement to guide the treatment of clinical infections.

## Introduction

*Enterobacter cloacae* complex (ECC) is widely spread in nature and is found in soil, sewage, and human gastrointestinal tract. It is one of the common opportunistic pathogens in hospitals, which leads to various infections, such as septicemia, pneumonia, urinary tract infections, and postsurgical peritonitis hospital-acquired infections (Wisplinghoff et al., 2004; Guerin et al., 2020). ECC produces a Bush class 1 beta-lactamase (AmpC) at a low level, conferring resistance to ampicillin, amoxicillin plus clavulanic acid, and cefoxitin (Hoffmann et al., 2005a). In recent years, with the widespread use and abuse of antibiotics, especially carbapenems, carbapenem-resistant ECC (CREC) has increased, showing a multidrug-resistance (MDR) phenotype, making these clinical infections a significant challenge (Annavajhala et al., 2019; Davin-Regli et al., 2019).

The ECC is a diverse bacterial population, and hence, it is crucial to identify the ECC group since it is a major pathogenic bacterium. Although this is a routine analysis based on phenotypic methods in clinical laboratories, the accurate identification of isolates within this taxon is challenging. In addition, the analysis of the *16S rRNA* gene is widely used for bacterial identification, but it has a poor discriminatory ability due to its 60% similarity among the subgroups in ECC (Hoffmann and Roggenkamp, 2003; Morand et al., 2009). Other previously described molecular methods to elucidate ECC included *rpoB* (Hoffmann and Roggenkamp, 2003; Case et al., 2007), MLSA (Paauw et al., 2008), microarray-based CGH (Paauw et al., 2008), the combination of MALDI-TOF MS with *E. cloacae*-specific duplex real-time polymerase chain reaction (PCR) (Pavlovic et al., 2012), and *hsp60* detection (Hoffmann and Roggenkamp, 2003; Morand et al., 2009; Guerin et al., 2016). Hoffmann and Roggenkamp (2003) showed that ECC could be divided into 12 genetic clusters (clusters I–XII) and one sequence crowd (XIII) based on the analysis of a fragment of the *hsp60* gene, known as the *groEL* homolog encoding a 60-kDa heat shock protein. The *hsp60* gene analysis method was optimal for distinguishing the subgroups clinically, as it was convenient and rapid. According to *hsp60* genotyping by Hoffmann and Roggenkamp (2003), the different clusters of the ECC are attributed to their nomenclature: cluster I (*Enterobacter asburiae*), cluster II (*Enterobacter kobei*), cluster III (*E. cloacae* III), cluster IV (*E. cloacae* IV), cluster V (*Enterobacter ludwigii*), cluster VI (*Enterobacter hormaechei* subsp. *oharae*), cluster VII (*E. hormaechei* subsp. *hormaechei*), cluster VIII (*E. hormaechei* subsp. *steigerwaltii*), cluster IX (*E. cloacae* IX), cluster X (*Enterobacter nimipressuralis*), cluster XI (*E. cloacae* subsp. *cloacae*), and cluster XII (*E. cloacae* subsp. *dissolvens*).

Some studies have reported the differences in the distribution and resistance against beta-lactams or colistin among the genetic clusters, including specific distribution of strains isolated from infected orthopedic implants (Morand et al., 2009), elective distribution of resistance to beta-lactams (Garinet et al., 2018), and cluster-dependent colistin hetero-resistance among ECC genetic clusters (Guerin et al., 2016). In addition, biofilm- and virulence-related genes also need to be studied. Biofilm is an aggregate of sessile microorganisms, which prevents the antibiotics from coming into contact with bacteria, leading to repeated infections. Also, virulence-related genes in ECC, such as siderophore and adhesin, play a specific role in its infection; these include *fimA* (type 1 fimbriae gene) (Brust et al., 2019), *mrkD* (type 3 fimbriae gene) (Compain et al., 2014), *csgD* (curli gene) (Brust et al., 2019), *papC* and *papD* (P pili genes) (Brust et al., 2019), *fyuA* (*Yersinia* siderophore receptor gene) (Johnson et al., 2015), *iroNec* (salmochelin siderophore receptor gene) (Johnson et al., 2015), *entB* (enterobactin gene) (May and Okabe, 2011), and *ybtS* (yersiniabactin gene) (El Fertas-Aissani et al., 2013).

However, the resistance to antibiotics, biofilm formation ability, and pathogenicity/virulence among the species of ECC have been yet unclear due to the limited data available in this research field. The present study aimed to explore the differences among the genetic clusters of ECC, providing theoretical guidance for the treatment of clinical infection.

## Materials and Methods

### Bacterial Isolates

A total of 130 isolates were randomly collected from the First Affiliated Hospital of Wenzhou Medical University (Wenzhou, China) in 2018. Antimicrobial susceptibility testing was performed using VITE^®^K2 (bioMérieux, Marcy-l’Étoile, France). Identification of all isolates was carried out using matrix-assisted laser desorption/ionization time-of-flight mass spectrometry (MALDI-TOF-MS) (bioMerieux, France). The isolates were stored in 30% glycerol at −80°C until further analysis. All protocols in this study were approved by the ethics committee of the First Affiliated Hospital of Wenzhou Medical University. Informed consent was waived because this observational design study focused mainly on bacteria and did not involve intervention to patients.

### Minimum Inhibitory Concentration Determination

According to the recent guidelines recommended by the Institute of Clinical and Laboratory Standards (CLSI), the minimum inhibitory concentrations (MICs) of 13 antimicrobial agents, including aztreonam (ATM), ciprofloxacin (CIP), levofloxacin (LVX), ceftriaxone (CRO), cefepime (FEP), ceftazidime (CAZ), imipenem (IPM), ertapenem (ETP), gentamicin (GEN), tobramycin (TOB), amikacin (AMK), trimethoprim-sulfamethoxazole (SXT), and nitrofurantoin (NIT), were determined by the agar dilution method. *Escherichia coli* ATCC 25922 was used as the control strain.

### Biofilm Formation Assay

To explore the differences in biofilm formation ability among different species of ECC, biofilm formation assays were performed in a 96-well polystyrene microtiter plate, as previously described with some modifications (Brust et al., 2019). Briefly, the isolates were grown overnight in Luria–Bertani (LB) broth at 37°C. Subsequently, the culture was adjusted to 0.5 McFarland with sterile normal saline, followed by 1:100 dilution in different media including trypticase soy broth (TSB), LB broth, LB broth with glucose (0.02 M), and M9 minimal medium supplemented with glucose (0.02 M) and MgSO_4_ (0.002 M). The microtiter plates were incubated at 37°C for 24 h. Then, the cell suspension was removed, and the plate was washed twice with 1× phosphate-buffered saline (PBS) and heat-fixed at 60°C for 1 h. An equivalent of 150 μl of 1% crystal violet solution (CV) was added to the wells for staining for 15 min. After excess CV was removed, the wells were washed three times with 1X PBS. Bound CV was solubilized in a 150-μl mixture of ethanol–acetic acid (95:5, *v*/*v*). The absorbance of CV was read at 595 nm on a microplate reader.

### Swimming Assay

To test the bacterial mobility among different species of ECC, swimming assays were carried out as described previously (Guerin et al., 2020), with some modifications. A volume of 2 μl of a log-phase culture was spotted on a TS semi-solid agar (0.5% agar) plate. The swimming zone was measured after incubation at 37°C overnight.

### Proteolytic Activity Assay

To test the bacterial proteolytic activity among various species of ECC, proteolytic activity assay was performed as described previously (Paudel et al., 2021). Eight strains of each cluster were randomly selected as experimental strains. Briefly, 2 μl of the overnight cultures were spotted on a milk agar plate and incubated at 37°C overnight. Subsequently, the proteolytic activity was determined by the appearance of a lysis loop surrounding the bacterial colonies.

### Detection of Virulence Determinants

In order to investigate the differences in virulence genes among various species of ECC, the genome DNA of all 130 ECC strains was extracted using the Biospin Bacterial Genomic DNA Extraction kit (Bioflux, Tokyo, Japan) according to the manufacturer’s instructions and served as templates for subsequent analysis. Virulence genes, including *fimA* (type 1 fimbriae gene) (Brust et al., 2019), *mrkD* (type 3 fimbriae gene) (Compain et al., 2014), *csgD* (curli gene) (Brust et al., 2019), *papC* and *papD* (P pili genes) (Brust et al., 2019), *fyuA* (*Yersinia* siderophore receptor gene) (Johnson et al., 2015), *iroNec* (salmochelin siderophore receptor gene) (Johnson et al., 2015), *entB* (enterobactin gene) (May and Okabe, 2011), and *ybtS* (yersiniabactin gene) (El Fertas-Aissani et al., 2013), were identified by PCR. The primers of all genes for PCR are listed in Supplementary Table 1. The products of PCR amplification were sequenced by Shanghai Genomics Institute Technology Co. Ltd. (Shanghai, China), and the sequencing data were analyzed using BLAST search against the NCBI database^1^.

### Identification Methods

Bacterial colonies were grown overnight at 37°C on a blood-agar plate and cultured in LB medium to the logarithmic growth phase. Then, bacterial DNA was extracted using the Biospin Bacterial Genomic DNA Extraction kit (Bioflux, Tokyo, Japan) according to the manufacturer’s instructions and served as templates for subsequent analysis. Partial sequencing of the *hsp60* gene was performed using a previously described protocol (Hoffmann and Roggenkamp, 2003). Briefly, oligonucleotide primers Hsp60-F (5′-GGTAGAAGAAGGCGTGGTTGC-3′) and Hsp60-R (5′-ATGCATTCGGTGGTGATCATCAG-3′) were used for genomic amplification of a 341-bp fragment of the *hsp60* gene. A negative control containing all reagents except the target DNA (which was replaced by H_2_O) was included in each series. PCR amplification was conducted on a GeneAmp PCR system 9700 apparatus (Applied Biosystems) for 30 cycles under the following conditions: denaturation at 94°C for 30 s, annealing at 57°C for 30 s, and elongation at 72°C for 60 s. The positive products of PCR amplification were sequenced by Shanghai Genomics Institute Technology. The sequencing data were analyzed using BLAST search against the NCBI database (see Text Footnote 1). The fragment of the *hsp60* gene was obtained for all 130 strains, and the sequence of the fragment was compared to the reference sequences of the strains described previously in taxonomic studies (Hoffmann and Roggenkamp, 2003) using the Clustal W algorithm. The sequence comparisons were exported as an unrooted circle neighbor-joining tree with proportional branch lengths.

### Evaluation of Virulence *in vivo* by the *Galleria mellonella* Infection Model

We evaluated six major clusters including I, II, III, VI, VIII, and IX. Then, eight strains from each of the clusters I, II, III, VI, VIII, and IX were selected randomly. The overnight cultures of ECC strains were washed with normal saline (NS) and further adjusted with NS to achieve the 0.5 McFarland standard. Insects weighing 250–350 mg were selected for the experiment, and those injected with NS were used as controls. Briefly, 10 μl of the 0.5 McFarland bacterial solution was injected into the rear left proleg of *G. mellonella* using a microinjector and incubated at 37°C. Subsequently, the survival rate of *G. mellonella* was recorded after 1, 2, 3, 4, 5, 6, and 7 days. Mortality rate and pathological changes in the vital organs were assessed by Kaplan–Meier analysis and log-rank test. Larvae were considered dead when they repeatedly failed to respond to physical stimuli. All experiments were conducted in triplicate.

### Nucleotide Sequence Accession Number

The sequences of the following type strains were retrieved from the GenBank database (the information in parentheses included strain designation, GenBank accession number): *E. asburiae* (ATCC 35953, AJ417141), *E. kobei* (ATCC BAA260, AJ567899), *E. cloacae* subsp. *dissolvens* (ATCC 23373, AJ417143), *E. cloacae* subsp. *cloacae* (ATCC 13049, AJ417142), *E. hormaechei* subsp. *steigerwaltii* (CIP108489, AJ543908), *E. hormaechei* subsp. *oharae* (EN-314, AJ543782), *E. nimipressuralis* (ATCC 9912, AJ567900), *E. hormaechei* subsp. *hormaechei* (ATCC 49162, AJ417108), *E. ludwigii* (EN-119, AJ417114), *E. cancerogenus* (ATCC 33241, AJ567895), *Enterobacter amnigenus* (ATCC 3072, AJ567894), *Cronobacter sakazaki* (ATCC 29544, AJ567902), *Enterobacter cowanii* (ATCC 107300T, AJ567896), *Enterobacter pyrinus* (ATCC 49851, AJ567901), *Enterobacter gergoviae* (ATCC 33028, AJ567897), and *Enterobacter aerogenes* (AB008141).

### Statistical Analysis

All data were analyzed using the GraphPad Prism v8.0.1 statistical software package (GraphPad Software, La Jolla, CA, United States). Unpaired Student’s *t*-test (two-tailed) was used to compare the ability of biofilm formation and mobility between different species of ECC. Chi-square test was used to compare the resistance rate among the species. Kaplan–Meier analysis and log-rank test were performed to analyze the survival rate of *G. mellonella* larvae. *P*-value < 0.05 indicated statistical significance.

## Results

### Predominance of *E. hormaechei* in *Enterobacter cloacae* Complex Clinical Isolates

**FIGURE 1:**
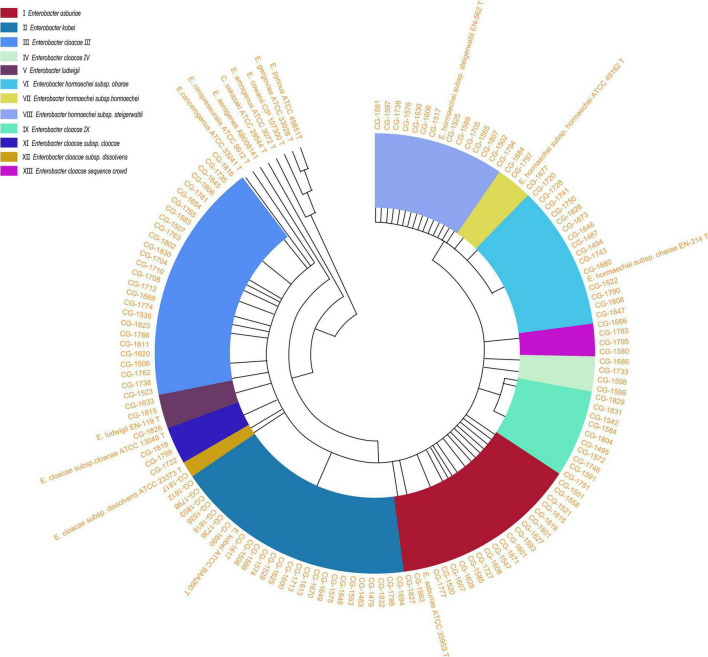
An unrooted circle neighbor-joining tree with proportional branch length resulting from analysis of the *hsp60* gene sequences of 130 clinical strains and previously reported sequences (Hoffmann and Roggenkamp, 2003; Hoffmann et al., 2005a,b; Iversen et al., 2008). The previously described strains correspond to sequences with GenBank accession numbers: *E. asburiae* ATCC 35853 T (AJ417141), *E. kobei* ATCC BAA260 T (AJ567899), *E. cloacae* subsp. *dissolvens* ATCC 23373 T (AJ417143), *E. cloacae* subsp. *cloacae* ATCC 13049 T (AJ417142), *E. hormaechei* subsp. *steigerwaltii* EN-562 T (AJ543908), *E. hormaechei* subsp. *oharae* EN-314 T (AJ543782), *E. nimipressuralis* ATCC 9912 T (AJ567900), *E. hormaechei* subsp. *hormaechei* ATCC 49162 T (AJ417108), *E. ludwigii* EN-119 T (AJ417114), *E. cancerogenus* ATCC 33241 T (AJ567895), *E. amnigenus* ATCC 3072 T (AJ567894), *C. sakazaki* ATCC 29544 T (AJ567902), *E. cowanii* ATCC 107300 T (AJ567896), *E. pyrinus* ATCC 49851 T (AJ567901), *E. gergoviae* ATCC 33028 T (AJ567897), and *E. aerogenes* AB008141 (AB008141). Genetic clusters are numbered according to the previous descriptions (Hoffmann and Roggenkamp, 2003). *E.*, *Enterobacter*.

**FIGURE 2:**
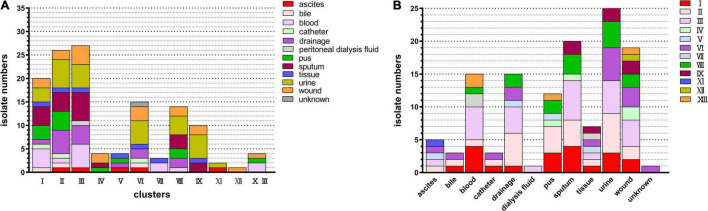
Distribution of clinical strains within the genetic clusters of ECC. All strains could be assigned to one of the previously reported *hsp60* gene sequencing-based genetic clusters of ECC. **(A)** Distribution of ECC isolates in clusters. Colored square indicates the source of the ECC strain. **(B)** Cluster distribution numbers for ECC isolates at various anatomical sites. Colored square indicates the clusters of the ECC strain. ECC, *Enterobacter cloacae* complex.

Partial sequencing analysis of the *hsp60* gene identified all isolates as part of the clusters that form the ECC (Figure 1). As shown in Figure 2A, 32/130 (24.6%) ECC strains belonged to the *E. hormaechei* species, including 15 *E. hormaechei* subsp. *oharae* (cluster VI), 3 *E. hormaechei* subsp. *hormaechei* (cluster VII), and 14 *E. hormaechei* subsp. *steigerwaltii* (cluster VIII). In addition, 27/130 (20.8%) strains belonged to cluster III, 26/130 (20%) strains belonged to *E. kobei* (cluster II), 20/130 (15.4%) strains belonged to *E. asburiae* (cluster I), 10/130 (7.7%) strains belonged to cluster IX, 4 strains in cluster IV, 4 strains in cluster V, 4 strains in cluster XIII, 2 strains in cluster XI (*E. cloacae* subsp. *cloacae*), and 1 strain in cluster XII (*E. cloacae* subsp. *dissolvens*). Cluster X (*E. nimipressuralis*) was absent in ECC isolates. However, MALDI-TOF-MS divided ECC into two species (9 *E. hormaechei* strains and 121 *E. cloacae* strains), with only 6.92% consistency rate with *hsp60* gene-based typing method (Supplementary Table 2).

As shown in Figure 2B, ECC was isolated from various sources, including ascites, bile, blood, catheter, drainage, peritoneal dialysis fluid, pus, sputum, tissue, urine, and wound. Among these, urine, sputum, and wound were the major separation sources, followed by blood (12 strains), drainage (8 strains), and pus (7 strains). The strains from the urine site consisted of clusters I, II, III, VI, VIII, and IX. Moreover, cluster III was the leading cluster in urine, wound, and sputum.

### Various Resistance Spectra of Strains in Different Clusters

**FIGURE 3:**
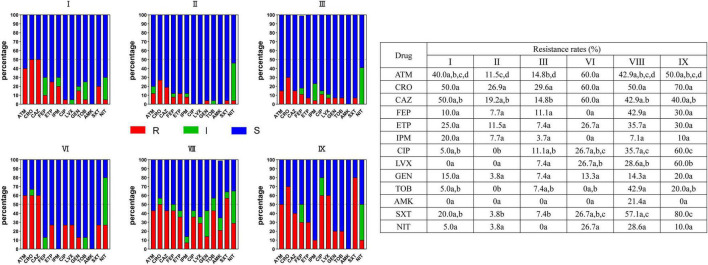
Resistance of strains in six major clusters I, II, III, VI, VIII, and IX to commonly used clinical antibacterial drugs in ECC strains. Red squares indicate drug resistance, green squares indicate intermediation, and blue squares indicate sensitivity. One or more identical letters of a, b, c, and d in different cells indicate no statistical difference of resistance rate, while different letters of a, b, c, and d among the cells indicate a statistical difference. For example, for aztreonam, there is no difference in the resistance rate between I and VIII, II and IX, II and III, but a difference in resistance rate is observed between II and VI. ECC, *Enterobacter cloacae* complex; R, resistance; I, intermediation; S, sensitivity.

As shown in Figure 3 and Supplementary Table 3, the resistance rates of the six major clusters were sorted out and analyzed. Together, the resistance to 13 antibiotics varied in different clusters. Clusters VIII (*E. hormaechei* subsp. *steigerwaltii*) and IX had high resistant rates to 13 antimicrobial agents, while *E. kobei* (cluster II) has a low resistant rate. For example, clusters VIII and IX had a higher resistance rate to ciprofloxacin (CIP) and levofloxacin (LVX) than cluster II (*P* < 0.05). The other clusters also showed differential resistance to these antimicrobial agents. Interestingly, the resistance of all clusters except cluster VIII was extremely low against amikacin.

### Stronger Biofilm Formation Ability of Cluster II Strains

**FIGURE 4:**
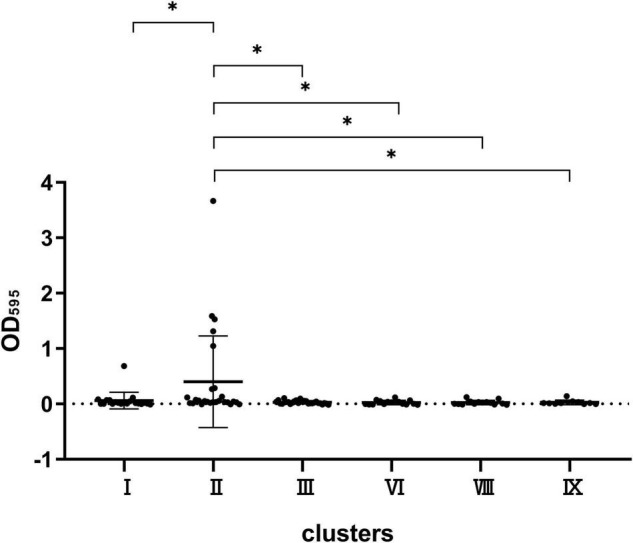
Comparison of the biofilm formation capacity of strains in clusters VIII, VI, III, II, I, and IX. *P < 0.05 means the biofilm formation capacity of clusters VIII, VI, III, I, and IX is significantly decreased compared to cluster II.

Herein, we compared the differences in biofilm formation ability of the six main types of clusters including I, II, III, VI, VIII, and IX. CV assay revealed that the six clusters did not differ significantly from each other when growing in TSB, LB, and 0.2 M LB media (data not shown). However, statistical differences were observed in the ability to form biofilm among the six clusters in M9 minimal medium supplemented with glucose (0.02 M) and MgSO_4_ (0.002 M) (Figure 4). The biofilm formation ability of cluster II was stronger than that of clusters I, III, VI, VIII, and IX, and no differences were observed among the other five clusters.

### Stronger Swimming Ability of Cluster I Strains

**FIGURE 5:**
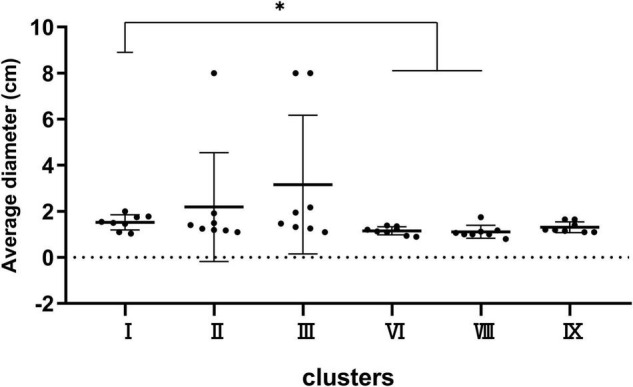
Bacterial mobility capacity of strains in clusters I, II, III, VI, VIII, and IX. *P < 0.05 means the swimming capacity of cluster I is significantly higher than clusters VI and VIII.

Strains were spotted on a TS semi-solid agar to observe the swimming ability of the six main clusters including I, II, III, VI, VIII, and IX. As shown in Figure 5, the mobility in cluster I was stronger than that in clusters VI and VIII (*P* < 0.05), while no significant difference was observed among any other clusters.

### High Proteolytic Activity of Cluster IX Strains

**FIGURE 6:**
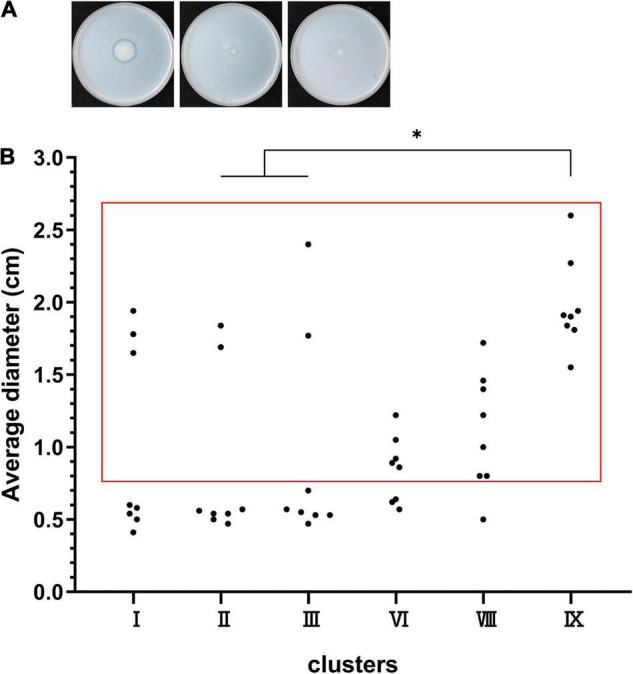
Protease production capacity of clusters I, II, III, VI, VIII, and IX. **(A)** The left panel shows the lysis loop of a high-protease-producing strain, the middle shows the lysis loop of a low-protease-producing strain, and the right shows no loop of a non-protease-producing strain. **(B)** The dots in the red box represent the protease-producing strains, and the dots outside the red box represent the non-protease-producing strains. *P < 0.05 means higher proteolytic activity of cluster IX compared to clusters II and III.

We randomly selected eight strains of clusters I, II, III, VI, VIII, and IX to test for protease production (Figure 6). The results displayed 8/8 protease-producing strains in cluster IX, 7/8 positive strains in cluster VIII, 5/8 positive strains in cluster VI, 3/8 positive strains in cluster I, 2/8 positive strains in cluster II, and 2/8 positive strains in cluster III (cluster IX vs. clusters II and III, *P* < 0.05) (Figure 6B). Notably, some great lysis loops were observed on plates (left panel in Figure 6A) for cluster IX strains, indicating high proteolytic activity.

### Fewer Virulence Genes Carried by Cluster II Strains

All strains were tested for virulence-related genes. As shown in Table 1, all strains carried fewer siderophore-related genes but many adhesin-related genes. Still, none of them carried *fyuA*, *entB*, and *ybtS* genes encoding siderophores or *mrkD* gene related to adhesin. For strains in cluster I, one of the six major clusters, the carrying rates of adhesin genes *fimA*, *csgD*, *papC*, and *papD* were 45, 5, 20, and 0%, respectively; and 3.85% *fimA*, 3.85% *csgD*, and no *papC* and *papD* for cluster II strains; 63% *fimA*, 29.6% *csgD*, 7.41% *papC*, and 3.7% *papD* for cluster III strains; 53.3% *fimA*, 46.7% *csgD*, 46.7% *papC*, and 53.3% *papD* for cluster VI strains; and 50% *fimA*, 35.7% *csgD*, 28.6% *papC*, and 42.9% *papD* for cluster VIII strains. Among these, cluster II strains carried fewer virulence-related genes at a low rate.

**TABLE 1:** Carrying rates of virulence-related genes between different clusters.

Clusters (*n*)	Virulence factors (%)								

	**Siderophores**				**Adhesins**				
	***fyuA***	***iroNec***	***entB***	***ybtS***	***fimA***	***csgD***	***papC***	***papD***	***mrkD***
I (*n* = 20)	0	0	0	0	45.0	5.00	20.0	0	0
II (*n* = 26)	0	3.85	0	0	3.85	3.85	0	0	0
III (*n* = 27)	0	3.70	0	0	63.0	29.6	7.41	3.70	0
IV (*n* = 4)	0	0	0	0	0	0	0	25.0	0
V (*n* = 4)	0	0	0	0	50.0	0	0	0	0
VI (*n* = 15)	0	6.67	0	0	53.3	46.7	46.7	53.3	0
VII (*n* = 3)	0	0	0	0	66.7	33.3	33.3	66.7	0
VIII (*n* = 14)	0	14.3	0	0	50.0	35.7	28.6	42.9	0
IX (*n* = 10)	0	0	0	0	90.0	60.0	30.0	90.0	0
XI (*n* = 2)	0	0	0	0	50.0	0	50.0	50.0	0
XII (*n* = 1)	0	0	0	0	0	100	0	0	0
XIII (*n* = 4)	0	25.0	0	0	75.0	75.0	50.0	50.0	0

### Differential Virulence *in vivo*

**FIGURE 7:**
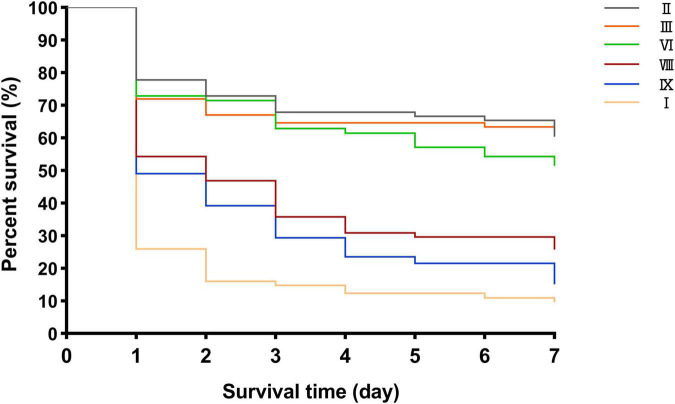
Different survival rates of *G. mellonella* infection model in clusters VIII, VI, III, II, I, and IX.

The results of the *G. mellonella* infection model showed that the virulence of cluster I strains was significantly stronger than that of the other five cluster strains, in the order of cluster IX, cluster VIII, cluster VI, cluster III, and cluster II strains (cluster VIII vs. one of the other five clusters, *P* < 0.05; cluster VIII vs. cluster IX, *P* > 0.05; cluster VIII vs. cluster VI, *P* < 0.05; cluster VI, cluster II, and cluster III showed no statistically significant differences) (Figure 7).

## Discussion

*Enterobacter cloacae* complex is one of the most common opportunistic nosocomial pathogens, leading to various infectious diseases, especially carbapenem-resistant strains. Although ECC is a complex flora composed of several species, such as *E. cloacae*, *E. asburiae*, *E. hormaechei*, *E. kobei*, *E. ludwigii*, *Enterobacter mori*, *and E. nimipressuralis*, clinicians treat them equally as “*E. cloacae.*” Other studies demonstrated differences in resistance among the species (Guerin et al., 2016; Garinet et al., 2018). Therefore, it is crucial to further identify the differences among the ECC subgroups to improve clinical treatments. In this study, we analyzed the distribution, drug resistance, biofilm formation ability, mobility, proteolytic activity, virulence-related gene carrying rate, and difference in virulence *in vivo* among strains in various clusters based on *hsp60* analysis.

The current study proved that all strains were divided into 12 genetic clusters (clusters I–XII) and one sequence crowd (sequence crowd XIII), except cluster X (*E. nimipressuralis*), consistent with the previous description of *E. nimipressuralis* isolated from non-clinical sources (Mezzatesta et al., 2012). In this study, *E. hormaechei* was the predominant species, which was different from the current understanding of the cloacae complex by “*E. cloacae*” but was in line with a previous study (Morand et al., 2009; Mateos et al., 2021). MALDI-TOF-MS divided ECC into two species and showed that *E. cloacae* was the main strain. The identification results of MALDI-TOF-MS and *hsp60* gene-based typing method were quite different with an only 6.92% consistency rate, which indicated the inability of MALDI-TOF-MS in distinguishing specific ECC species. Therefore, there is an urgent need for more accurate routine bacterial identification tools in clinical practice. The most common ECC infection site was urine, consistent with the finding by Mateos et al. (2021).

Our results indicated that the drug resistance of strains in different clusters varied among ECC subgroups, which was similar to the previous report (Mateos et al., 2021). Moreover, cluster VIII and IX strains had a high resistance rate to common clinical antibacterial drugs. Nonetheless, the resistance rate of all six clusters to amikacin was zero, except for cluster VIII. As reported previously, ECC remained highly sensitive to amikacin (Davin-Regli and Pages, 2015; Mateos et al., 2021). Most ECC isolates were susceptible to fluoroquinolones, trimethoprim/sulfamethoxazole, chloramphenicol, aminoglycosides, tetracyclines, piperacillin–tazobactam, and carbapenems, while intrinsically resistant to ampicillin, amoxicillin, amoxicillin–clavulanate, first-generation cephalosporins, and cefoxitin, owing to constitutive AmpC β-lactamase (Mezzatesta et al., 2012). However, some strains were still resistant to the first-line drugs, such as carbapenem and amikacin. In other Enterobacteriaceae such as *Klebsiella*, the China Bacterial Resistance Surveillance Network^2^ (CHINET, 2021) shows *Klebsiella* has higher resistant rates to commonly used antibacterial drugs, such as amikacin, fluoroquinolones, tetracyclines, and carbapenem. The carbapenem resistance has been attributed to carbapenemase or the production of AmpC or ESBLs combined with decreased expression of membrane protein (Liu et al., 2021). The aminoglycoside resistance has been ascribed to the presence of a plasmid encoding aminoglycoside-modifying enzymes (Davin-Regli et al., 2019).

In addition to the differential resistance spectra of various cluster strains, we also explored the ability of biofilm formation among the strains in six clusters. Hence, different clusters were inoculated in varying nutrient media to form biofilms. Interestingly, cluster II strain had the most potent film formation ability in a nutrient-deficient M9 medium, indicating that the bacteria could grow and survive without nutrition. This corresponded to bacterial invasion into the urine as discussed above. These results further demonstrate that ECC is an opportunistic pathogen, which can easily infect severely ill patients with weakened immunity (Nepal et al., 2021). Under such malnutrition conditions, the strong biofilm production capacity of cluster II increases the possibility of its invasion into deep parts of the human body. In addition, other Enterobacteriaceae such as *Klebsiella pneumoniae* could also form biofilms in M9 medium (Amaretti et al., 2020).

Our results showed differences in the mobility between different cluster strains, which was in line with their biochemical characteristics (Mezzatesta et al., 2012). Strains with strong mobility, such as cluster I species, showed high pathogenicity. In addition to mobility, protease production was also one of the characteristics of bacterial virulence (Shao et al., 2018). Additionally, 100% (8/8) cluster IX strains produced protease and had high protease activity, indicating high pathogenicity.

The main virulence genes carried by ECC were *fimA* and *csgD*, while in other Enterobacteriaceae, such as *Klebsiella pneumoniae*, the main virulence genes were *mrkD* and *entB* (Amaretti et al., 2020). The carrying rates of virulence-related genes *in vivo* proved that the virulence and pathogenicity of cluster II strains were weak. However, why cluster II strains are common in clinical isolates is yet to be elucidated. Herein, we speculated that they might have a strong biofilm formation ability. Clusters VIII and I strains had high pathogenicity, which explained why clinically isolated strains accounted for a large proportion of this organism. Thus, the characteristics of strains in different types of the clusters varied. Similarly, *K. pneumoniae* complexes were divided into seven phylogenetic groups (Kp1 to Kp7) based on whole-genome sequencing and recombination-purged nucleotide sequence alignments of 1,703 core genes or by sequencing taxonomic marker genes such as *rpoB* (Rodrigues et al., 2019). The members of these *K. pneumoniae* complexes differed in source distribution, drug resistance, and virulence (Brisse et al., 2004; Holt et al., 2015; Long et al., 2017; Janssen et al., 2020). However, all members of this species complex were misidentified as *K. pneumoniae* or *Klebsiella variicola* using standard laboratory methods, such as MALDI-TOF-MS, which masked their actual clinical and epidemiological significance (Brisse et al., 2004; Seki et al., 2013; Long et al., 2017). Hence, intensive focus by clinicians and an efficient method to identify various bacterial subgroups are required.

## Conclusion

The characteristics of resistance, biofilm-forming ability, mobility, and virulence were different among strains in various clusters. The main clusters of ECC clinical strains were I, II, III, VI, VIII, and IX, of which I, VIII, and IX were predominant with high resistance and pathogenicity, and cluster II (*E. kobei*) was a special taxon with strong biofilm formation ability under nutrient-deficient conditions but associated with low resistance, virulence, and pathogenicity. Hence, clinical classification methods to identify the subgroups of ECC are urgently needed to guide the treatment of clinical infections.

## Data Availability Statement

The original contributions presented in the study are included in the article/Supplementary Material, further inquiries can be directed to the corresponding authors.

## Author Contributions

SL conducted the experiments, analyzed the data, and wrote the manuscript. LC participated in the experiments and writing. LW and BZ provided isolates and analyzed the data. DY and XZ participated in the analysis of the results. YL and WZ revised the manuscript. JY and TZ helped to design the study. All authors read and approved the manuscript. All authors contributed to the article and approved the submitted version.

## Conflict of Interest

The authors declare that the research was conducted in the absence of any commercial or financial relationships that could be construed as a potential conflict of interest. The reviewer BZ declared a shared affiliation with one of the authors, JY to the handling editor at the time of the review.

## Publisher’s Note

All claims expressed in this article are solely those of the authors and do not necessarily represent those of their affiliated organizations, or those of the publisher, the editors and the reviewers. Any product that may be evaluated in this article, or claim that may be made by its manufacturer, is not guaranteed or endorsed by the publisher.

## Footnotes

1
www.ncbi.nlm.nih.gov/BLAST

2
http://www.chinets.com/
