# Supplementary material for: Cluster Differences in Antibiotic Resistance, Biofilm Formation, Mobility, and Virulence of Clinical Enterobacter cloacae Complex
Source: Front Microbiol. 2022 Apr 6;13:814831. doi: 10.3389/fmicb.2022.814831 (PMC9019753; doi:10.3389/fmicb.2022.814831)
Supplement: Supplementary file 1 [file Table_1.docx]

**TABLE S1 |** Primer sequence, production size and annealing temperature used in this study.

| **Primer** | **Suquence** | **Size (bp)** | **Annealing (℃)** | **references** |
| --- | --- | --- | --- | --- |
| *csgD* | **F: CCTTCCTTACAAGCGACAGC**  **R: TCGCGGAAAGGATACTCATC** | 236 | 58 | (Brust et al., 2019) |
| *fimA* | **F: TGCTGTCGAGGATCTCAATG**  **R:ACGGTTAATCTCGGCCAGTA** | 229 | 58 | (Brust et al., 2019) |
| *fyuA* | **F: TGATTAACCCCGCGACGGGAA**  **R: CGCAGTAGGCACGATGTTGTA** | 787 | 55 | (Johnson et al., 2015) |
| *iroNec* | **F: AAGTCAAAGCAGGGGTTGCCCG**  **R: GACGCCGACATTAAGACGCAG** | 667 | 55 | (Johnson et al., 2015) |
| *papC* | **F: CCCTGAAGACCGATGACAAT**  **R: CGGAACGGAGGTTTGATAGA** | 148 | 58 | (Brust et al., 2019) |
| *papD* | **F: TGGATGGAAGACGAGAAAGG**  **R: CATCCAGTACAGCGTCTCG** | 134 | 58 | (Brust et al., 2019) |
| *ybtS* | **F**: **AGTGGTGCGTTCTGCGTC**  **R**: **ATTTCTACATCTGGCGTTA** | 477 | 50 | (El Fertas-Aissani et al., 2013) |

Brust, F.R., Boff, L., da Silva Trentin, D., Pedrotti Rozales, F., Barth, A.L., and Macedo, A.J. (2019). Macrocolony of NDM-1 Producing Enterobacter hormaechei subsp. oharae Generates Subpopulations with Different Features Regarding the Response of Antimicrobial Agents and Biofilm Formation. *Pathogens* 8(2). doi: 10.3390/pathogens8020049.

El Fertas-Aissani, R., Messai, Y., Alouache, S., and Bakour, R. (2013). Virulence profiles and antibiotic susceptibility patterns of Klebsiella pneumoniae strains isolated from different clinical specimens. *Pathol Biol (Paris)* 61(5)**,** 209-216. doi: 10.1016/j.patbio.2012.10.004.

Johnson, J.R., Porter, S., Johnston, B., Kuskowski, M.A., Spurbeck, R.R., Mobley, H.L., et al. (2015). Host Characteristics and Bacterial Traits Predict Experimental Virulence for Escherichia coli Bloodstream Isolates From Patients With Urosepsis. *Open Forum Infect Dis* 2(3)**,** ofv083. doi: 10.1093/ofid/ofv083.
