# Supplementary material for: Cluster Differences in Antibiotic Resistance, Biofilm Formation, Mobility, and Virulence of Clinical Enterobacter cloacae Complex
Source: Front Microbiol. 2022 Apr 6;13:814831. doi: 10.3389/fmicb.2022.814831 (PMC9019753; doi:10.3389/fmicb.2022.814831)
Supplement: Supplementary file 2 [file Table_2.docx]

**TABLE S2** **|** Identification of 130 ECC Isolates by Based on *hsp60* Analysis and MALDI-TOF-MS.

| **Isolates** | **Based on *hsp60* analysis** | | **MALDI-TOF-MS** | **Consistency rate (%)** |
| --- | --- | --- | --- | --- |
|  | **Clusters** | **Species** | **Species** |  |
| CG1501 | Ⅰ | *E. asburiae* | *E. cloacae* | 6.92% (9/130) |
| CG1520 | Ⅰ | *E. asburiae* | *E. cloacae* |  |
| CG1521 | Ⅰ | *E. asburiae* | *E. hormaechei* |  |
| CG1547 | Ⅰ | *E. asburiae* | *E. cloacae* |  |
| CG1558 | Ⅰ | *E. asburiae* | *E. cloacae* |  |
| CG1563 | Ⅰ | *E. asburiae* | *E. cloacae* |  |
| CG1585 | Ⅰ | *E. asburiae* | *E. cloacae* |  |
| CG1593 | Ⅰ | *E. asburiae* | *E. cloacae* |  |
| CG1601 | Ⅰ | *E. asburiae* | *E. cloacae* |  |
| CG1607 | Ⅰ | *E. asburiae* | *E. cloacae* |  |
| CG1608 | Ⅰ | *E. asburiae* | *E. cloacae* |  |
| CG1615 | Ⅰ | *E. asburiae* | *E. cloacae* |  |
| CG1619 | Ⅰ | *E. asburiae* | *E. cloacae* |  |
| CG1627 | Ⅰ | *E. asburiae* | *E. cloacae* |  |
| CG1628 | Ⅰ | *E. asburiae* | *E. cloacae* |  |
| CG1671 | Ⅰ | *E. asburiae* | *E. cloacae* |  |
| CG1727 | Ⅰ | *E. asburiae* | *E. cloacae* |  |
| CG1777 | Ⅰ | *E. asburiae* | *E. cloacae* |  |
| CG1801 | Ⅰ | *E. asburiae* | *E. cloacae* |  |
| CG1827 | Ⅰ | *E. asburiae* | *E. cloacae* |  |
| CG1479 | Ⅱ | *E. kobei* | *E. cloacae* |  |
| CG1483 | Ⅱ | *E. kobei* | *E. cloacae* |  |
| CG1508 | Ⅱ | *E. kobei* | *E. cloacae* |  |
| CG1528 | Ⅱ | *E. kobei* | *E. cloacae* |  |
| CG1538 | Ⅱ | *E. kobei* | *E. cloacae* |  |
| CG1553 | Ⅱ | *E. kobei* | *E. cloacae* |  |
| CG1574 | Ⅱ | *E. kobei* | *E. cloacae* |  |
| CG1575 | Ⅱ | *E. kobei* | *E. cloacae* |  |
| CG1588 | Ⅱ | *E. kobei* | *E. cloacae* |  |
| CG1600 | Ⅱ | *E. kobei* | *E. cloacae* |  |
| CG1612 | Ⅱ | *E. kobei* | *E. cloacae* |  |
| CG1613 | Ⅱ | *E. kobei* | *E. cloacae* |  |
| CG1617 | Ⅱ | *E. kobei* | *E. cloacae* |  |
| CG1625 | Ⅱ | *E. kobei* | *E. cloacae* |  |
| CG1648 | Ⅱ | *E. kobei* | *E. cloacae* |  |
| CG1649 | Ⅱ | *E. kobei* | *E. cloacae* |  |
| CG1670 | Ⅱ | *E. kobei* | *E. cloacae* |  |
| CG1690 | Ⅱ | *E. kobei* | *E. cloacae* |  |
| CG1694 | Ⅱ | *E. kobei* | *E. cloacae* |  |
| CG1713 | Ⅱ | *E. kobei* | *E. cloacae* |  |
| CG1736 | Ⅱ | *E. kobei* | *E. cloacae* |  |
| CG1788 | Ⅱ | *E. kobei* | *E. cloacae* |  |
| CG1798 | Ⅱ | *E. kobei* | *E. cloacae* |  |
| CG1803 | Ⅱ | *E. kobei* | *E. cloacae* |  |
| CG1818 | Ⅱ | *E. kobei* | *E. cloacae* |  |
| CG1832 | Ⅱ | *E. kobei* | *E. cloacae* |  |
| CG1506 | Ⅲ | *E. cloacae* Ⅲ | *E. cloacae* |  |
| CG1507 | Ⅲ | *E. cloacae* Ⅲ | *E. cloacae* |  |
| CG1523 | Ⅲ | *E. cloacae* Ⅲ | *E. cloacae* |  |
| CG1535 | Ⅲ | *E. cloacae* Ⅲ | *E. cloacae* |  |
| CG1583 | Ⅲ | *E. cloacae* Ⅲ | *E. cloacae* |  |
| CG1611 | Ⅲ | *E. cloacae* Ⅲ | *E. cloacae* |  |
| CG1620 | Ⅲ | *E. cloacae* Ⅲ | *E. cloacae* |  |
| CG1645 | Ⅲ | *E. cloacae* Ⅲ | *E. cloacae* |  |
| CG1654 | Ⅲ | *E. cloacae* Ⅲ | *E. cloacae* |  |
| CG1669 | Ⅲ | *E. cloacae* Ⅲ | *E. cloacae* |  |
| CG1704 | Ⅲ | *E. cloacae* Ⅲ | *E. cloacae* |  |
| CG1708 | Ⅲ | *E. cloacae* Ⅲ | *E. cloacae* |  |
| CG1710 | Ⅲ | *E. cloacae* Ⅲ | *E. cloacae* |  |
| CG1712 | Ⅲ | *E. cloacae* Ⅲ | *E. cloacae* |  |
| CG1735 | Ⅲ | *E. cloacae* Ⅲ | *E. cloacae* |  |
| CG1738 | Ⅲ | *E. cloacae* Ⅲ | *E. cloacae* |  |
| CG1761 | Ⅲ | *E. cloacae* Ⅲ | *E. cloacae* |  |
| CG1762 | Ⅲ | *E. cloacae* Ⅲ | *E. cloacae* |  |
| CG1763 | Ⅲ | *E. cloacae* Ⅲ | *E. cloacae* |  |
| CG1765 | Ⅲ | *E. cloacae* Ⅲ | *E. cloacae* |  |
| CG1766 | Ⅲ | *E. cloacae* Ⅲ | *E. cloacae* |  |
| CG1774 | Ⅲ | *E. cloacae* Ⅲ | *E. cloacae* |  |
| CG1802 | Ⅲ | *E. cloacae* Ⅲ | *E. cloacae* |  |
| CG1806 | Ⅲ | *E. cloacae* Ⅲ | *E. cloacae* |  |
| CG1816 | Ⅲ | *E. cloacae* Ⅲ | *E. cloacae* |  |
| CG1823 | Ⅲ | *E. cloacae* Ⅲ | *E. cloacae* |  |
| CG1830 | Ⅲ | *E. cloacae* Ⅲ | *E. cloacae* |  |
| CG1598 | Ⅳ | *E. cloacae* Ⅳ | *E. hormaechei* |  |
| CG1599 | Ⅳ | *E. cloacae* Ⅳ | *E. cloacae* |  |
| CG1686 | Ⅳ | *E. cloacae* Ⅳ | *E. cloacae* |  |
| CG1733 | Ⅳ | *E. cloacae* Ⅳ | *E. cloacae* |  |
| CG1633 | Ⅴ | *E. ludwigii* | *E. cloacae* |  |
| CG1722 | Ⅴ | *E. ludwigii* | *E. cloacae* |  |
| CG1815 | Ⅴ | *E. ludwigii* | *E. cloacae* |  |
| CG1826 | Ⅴ | *E. ludwigii* | *E. cloacae* |  |
| **CG1487** | **Ⅵ** | ***E. hormaechei subsp. oharae*** | ***E. hormaechei*** |  |
| CG1494 | Ⅵ | *E. hormaechei subsp. oharae* | *E. cloacae* |  |
| CG1522 | Ⅵ | *E. hormaechei subsp. oharae* | *E. cloacae* |  |
| CG1646 | Ⅵ | *E. hormaechei subsp. oharae* | *E. cloacae* |  |
| CG1647 | Ⅵ | *E. hormaechei subsp. oharae* | *E. cloacae* |  |
| CG1673 | Ⅵ | *E. hormaechei subsp. oharae* | *E. cloacae* |  |
| CG1680 | Ⅵ | *E. hormaechei subsp. oharae* | *E. cloacae* |  |
| CG1720 | Ⅵ | *E. hormaechei subsp. oharae* | *E. cloacae* |  |
| CG1728 | Ⅵ | *E. hormaechei subsp. oharae* | *E. cloacae* |  |
| CG1741 | Ⅵ | *E. hormaechei subsp. oharae* | *E. cloacae* |  |
| CG1743 | Ⅵ | *E. hormaechei subsp. oharae* | *E. cloacae* |  |
| CG1750 | Ⅵ | *E. hormaechei subsp. oharae* | *E. cloacae* |  |
| CG1790 | Ⅵ | *E. hormaechei subsp. oharae* | *E. cloacae* |  |
| CG1808 | Ⅵ | *E. hormaechei subsp. oharae* | *E. cloacae* |  |
| CG1828 | Ⅵ | *E. hormaechei subsp. oharae* | *E. cloacae* |  |
| CG1677 | Ⅶ | *E. hormaechei subsp. hormaechei* | *E. cloacae* |  |
| **CG1684** | **Ⅶ** | ***E. hormaechei subsp. hormaechei*** | ***E. hormaechei*** |  |
| CG1797 | Ⅶ | *E. hormaechei subsp. hormaechei* | *E. cloacae* |  |
| CG1502 | Ⅷ | *E. hormaechei subsp. steigerwaltii* | *E. cloacae* |  |
| CG1517 | Ⅷ | *E. hormaechei subsp. steigerwaltii* | *E. cloacae* |  |
| **CG1525** | **Ⅷ** | ***E. hormaechei subsp. steigerwaltii*** | ***E. hormaechei*** |  |
| **CG1530** | **Ⅷ** | ***E. hormaechei subsp. steigerwaltii*** | ***E. hormaechei*** |  |
| CG1565 | Ⅷ | *E. hormaechei subsp. steigerwaltii* | *E. cloacae* |  |
| CG1576 | Ⅷ | *E. hormaechei subsp. steigerwaltii* | *E. cloacae* |  |
| CG1581 | Ⅷ | *E. hormaechei subsp. steigerwaltii* | *E. cloacae* |  |
| CG1589 | Ⅷ | *E. hormaechei subsp. steigerwaltii* | *E. cloacae* |  |
| CG1597 | Ⅷ | *E. hormaechei subsp. steigerwaltii* | *E. cloacae* |  |
| CG1606 | Ⅷ | *E. hormaechei subsp. steigerwaltii* | *E. cloacae* |  |
| **CG1705** | **Ⅷ** | ***E. hormaechei subsp. steigerwaltii*** | ***E. hormaechei*** |  |
| CG1739 | Ⅷ | *E. hormaechei subsp. steigerwaltii* | *E. cloacae* |  |
| CG1794 | Ⅷ | *E. hormaechei subsp. steigerwaltii* | *E. cloacae* |  |
| **CG1807** | **Ⅷ** | ***E. hormaechei subsp. steigerwaltii*** | ***E. hormaechei*** |  |
| CG1495 | Ⅸ | *E. cloacae* Ⅸ | *E. cloacae* |  |
| CG1542 | Ⅸ | *E. cloacae* Ⅸ | *E. cloacae* |  |
| CG1572 | Ⅸ | *E. cloacae* Ⅸ | *E. cloacae* |  |
| CG1584 | Ⅸ | *E. cloacae* Ⅸ | *E. hormaechei* |  |
| CG1591 | Ⅸ | *E. cloacae* Ⅸ | *E. cloacae* |  |
| CG1746 | Ⅸ | *E. cloacae* Ⅸ | *E. cloacae* |  |
| CG1751 | Ⅸ | *E. cloacae* Ⅸ | *E. cloacae* |  |
| CG1804 | Ⅸ | *E. cloacae* Ⅸ | *E. cloacae* |  |
| CG1829 | Ⅸ | *E. cloacae* Ⅸ | *E. cloacae* |  |
| CG1831 | Ⅸ | *E. cloacae* Ⅸ | *E. cloacae* |  |
| **CG1799** | **Ⅺ** | ***E. cloacae subsp. cloacae*** | ***E. cloacae*** |  |
| **CG1819** | **Ⅺ** | ***E. cloacae subsp. cloacae*** | ***E. cloacae*** |  |
| **CG1817** | **Ⅻ** | ***E. cloacae subsp. dissolvens*** | ***E. cloacae*** |  |
| CG1580 | ⅩⅢ | sequence crowd XIII | *E. cloacae* |  |
| CG1666 | ⅩⅢ | sequence crowd XIII | *E. cloacae* |  |
| CG1783 | ⅩⅢ | sequence crowd XIII | *E. cloacae* |  |
| CG1795 | ⅩⅢ | sequence crowd XIII | *E. cloacae* |  |

Annotation: Bold indicates that the identification results of the two methods were consistent.
