# Supplementary material for: Cluster Differences in Antibiotic Resistance, Biofilm Formation, Mobility, and Virulence of Clinical Enterobacter cloacae Complex
Source: Front Microbiol. 2022 Apr 6;13:814831. doi: 10.3389/fmicb.2022.814831 (PMC9019753; doi:10.3389/fmicb.2022.814831)
Supplement: Supplementary file 3 [file Table_3.docx]

**TABLE S3** **|** Minimum Inhibitory Concentrations (MICs) of 130 ECC Isolates.

| **Isolates** | **MIC(μg/mL)** | | | | | | | | | | | | |
| --- | --- | --- | --- | --- | --- | --- | --- | --- | --- | --- | --- | --- | --- |
|  | **ATM** | **CRO** | **CAZ** | **FEP** | **ETP** | **IPM** | **CIP** | **LVX** | **GEN** | **TOB** | **AMK** | **SXT** | **NIT** |
| CG1479 | >32 | >32 | >32 | 4 | >=2 | >=4 | <=0.25 | <=0.25 | <=1 | <=1 | <=2 | <=20 | 32 |
| CG1483 | 32 | >32 | >32 | <=1 | <=0.5 | <=1 | <=0.25 | <=0.25 | <=1 | <=1 | <=2 | <=20 | 32 |
| CG1487 | <=4 | <=1 | <=4 | <=2 | <=0.5 | <=1 | <=0.25 | <=0.5 | <=4 | <=4 | <=16 | <=40 | <=32 |
| CG1494 | 32 | >32 | >32 | <=1 | 4 | <=1 | >2 | >4 | <=1 | <=1 | <=2 | >160 | 128 |
| CG1495 | <=1 | 8 | <=1 | <=1 | <=0.5 | <=1 | 2 | 1 | <=1 | <=1 | <=2 | >160 | <=16 |
| CG1501 | <=1 | <=1 | <=1 | <=1 | <=0.5 | <=1 | 1 | 1 | <=1 | <=1 | <=2 | <=20 | 32 |
| CG1502 | <=1 | <=1 | <=1 | <=1 | <=0.5 | <=1 | >2 | >4 | <=1 | <=1 | <=2 | >160 | 64 |
| CG1506 | >32 | >32 | >32 | 4 | >4 | >8 | 1 | 1 | >8 | >8 | 16 | <=20 | <=16 |
| CG1507 | <=1 | <=1 | <=1 | <=1 | <=0.5 | <=1 | <=0.25 | 00.5 | <=1 | <=1 | <=2 | <=20 | 64 |
| CG1508 | 8 | 16 | 32 | <=1 | <=0.5 | <=1 | <=0.25 | <=0.25 | <=1 | <=1 | <=2 | <=20 | 32 |
| CG1517 | <=1 | <=1 | <=1 | <=1 | <=0.5 | <=1 | <=0.25 | <=0.25 | 2 | 2 | 16 | <=20 | <=16 |
| CG1520 | 32 | >32 | >32 | <=1 | <=0.5 | <=1 | <=0.25 | <=0.25 | <=1 | <=1 | <=2 | <=20 | 32 |
| CG1521 | >=16 | >=4 | >=16 | 4 | <=0.5 | <=1 | <=0.25 | <=0.5 | >=16 | >=16 | <=16 | >=80 | <=32 |
| CG1522 | >32 | >32 | >32 | 8 | 2 | <=1 | <=0.25 | <=0.25 | <=1 | <=1 | <=2 | <=20 | 256 |
| CG1523 | <=1 | <=1 | <=1 | <=1 | <=0.5 | <=1 | <=0.25 | <=0.25 | <=1 | <=1 | <=2 | <=20 | <=16 |
| CG1525 | <=4 | <=1 | <=4 | <=2 | <=0.5 | <=1 | <=0.25 | <=0.5 | <=4 | <=4 | <=16 | <=40 | 64 |
| CG1528 | <=1 | <=1 | <=1 | <=1 | <=0.5 | <=1 | <=0.25 | <=0.25 | <=1 | <=1 | <=2 | <=20 | <=16 |
| CG1530 | >=16 | >=4 | >=16 | >32 | >=2 | <=1 | <=0.25 | <=0.5 | 8 | >=16 | 32 | >=80 | 64 |
| CG1535 | <=1 | <=1 | <=1 | <=1 | <=0.5 | <=1 | <=0.25 | 00.5 | <=1 | <=1 | <=2 | <=20 | 32 |
| CG1538 | <=1 | <=1 | <=1 | <=1 | <=0.5 | <=1 | <=0.25 | <=0.25 | <=1 | <=1 | <=2 | <=20 | 32 |
| CG1542 | >32 | >32 | >32 | >32 | <=0.5 | <=1 | >2 | >4 | >8 | >8 | 4 | >160 | 64 |
| CG1547 | >32 | >32 | >32 | >32 | >4 | >=4 | >2 | 4 | <=1 | <=1 | 4 | >160 | 128 |
| CG1553 | <=1 | <=1 | <=1 | <=1 | <=0.5 | <=1 | <=0.25 | <=0.25 | <=1 | <=1 | <=2 | <=20 | 64 |
| CG1558 | <=1 | <=1 | <=1 | <=1 | <=0.5 | <=1 | <=0.25 | 00.5 | 8 | 8 | <=2 | <=20 | 32 |
| CG1563 | — | >=4 | >=16 | 4 | >=2 | <=1 | <=0.25 | — | <=4 | — | <=16 | <=40 | <=32 |
| CG1565 | >32 | >32 | >32 | 32 | >=2 | <=1 | 2 | 2 | 8 | >8 | 32 | >160 | 64 |
| CG1572 | <=1 | <=1 | <=1 | <=1 | <=0.5 | <=1 | >2 | >4 | <=1 | <=1 | <=2 | >160 | <=16 |
| CG1574 | 2 | >32 | >32 | 32 | 4 | >=4 | 1 | 1 | <=1 | <=1 | <=2 | <=20 | 64 |
| CG1575 | <=1 | <=1 | <=1 | <=1 | <=0.5 | <=1 | <=0.25 | <=0.25 | <=1 | <=1 | <=2 | <=20 | 64 |
| CG1576 | <=1 | <=1 | <=1 | <=1 | <=0.5 | <=1 | >2 | 4 | <=1 | 8 | <=2 | >160 | 128 |
| CG1580 | <=1 | <=1 | <=1 | <=1 | <=0.5 | <=1 | <=0.25 | <=0.25 | <=1 | <=1 | <=2 | <=20 | 64 |
| CG1581 | >32 | >32 | >32 | 32 | 2 | <=1 | >2 | >4 | 8 | >8 | 16 | >160 | 128 |
| CG1583 | <=1 | <=1 | <=1 | <=1 | <=0.5 | <=1 | 1 | 1 | <=1 | <=1 | <=2 | <=20 | 64 |
| CG1584 | >=16 | >=4 | >=16 | 4 | >=2 | <=1 | >=1 | >=2 | <=4 | <=4 | <=16 | >=80 | >=128 |
| CG1585 | <=1 | <=1 | <=1 | <=1 | <=0.5 | <=1 | <=0.25 | <=0.25 | <=1 | <=1 | <=2 | <=20 | 32 |
| CG1588 | 2 | <=1 | <=1 | <=1 | <=0.5 | <=1 | <=0.25 | <=0.25 | <=1 | <=1 | <=2 | <=20 | 64 |
| CG1589 | >32 | >32 | >32 | 4 | 1 | <=1 | >2 | >4 | 4 | >8 | >32 | >160 | 256 |
| CG1591 | >32 | >32 | >32 | >32 | >4 | >=4 | >2 | >4 | <=4 | - | <=16 | >160 | <=16 |
| CG1593 | <=4 | >32 | >32 | >=16 | >=2 | >=4 | 00.5 | 1 | <=1 | 8 | <=2 | <=20 | <=16 |
| CG1597 | <=4 | 2 | <=4 | <=2 | <=0.5 | <=1 | <=0.25 | <=0.5 | >=16 | >=16 | >32 | >=80 | <=32 |
| CG1598 | <=1 | 2 | <=1 | <=1 | <=0.5 | <=1 | 00.5 | 00.5 | >8 | >8 | >32 | 40 | <=16 |
| CG1599 | <=1 | <=1 | <=1 | <=1 | <=0.5 | <=1 | <=0.25 | <=0.25 | <=1 | <=1 | <=2 | <=20 | 64 |
| CG1600 | - | >32 | >32 | <=1 | 00.25 | <=0.25 | - | <=.12 | - | - | <=2 | <=20 | - |
| CG1601 | 16 | >32 | 4 | >=16 | <=0.5 | 2 | 2 | 1 | >8 | 8 | <=2 | >160 | 64 |
| CG1606 | >32 | >32 | >32 | >32 | >=2 | 2 | >2 | >4 | >8 | >8 | >32 | >160 | 256 |
| CG1607 | 32 | >32 | >32 | <=1 | <=0.5 | <=1 | <=0.25 | <=0.25 | <=1 | <=1 | <=2 | <=20 | 32 |
| CG1608 | 16 | >32 | >32 | 4 | >4 | >=4 | 1 | 1 | >8 | 8 | <=2 | <=20 | 64 |
| CG1611 | <=1 | <=1 | <=1 | <=1 | <=0.5 | <=1 | 1 | 1 | <=1 | <=1 | <=2 | <=20 | 64 |
| CG1612 | <=1 | <=1 | <=1 | <=1 | <=0.5 | <=1 | <=0.25 | <=0.25 | <=1 | <=1 | <=2 | <=20 | 32 |
| CG1613 | 8 | 8 | <=2 | <=4 | >=2 | <=1 | <=0.25 | <=0.25 | <=1 | <=1 | <=2 | <=20 | 64 |
| CG1615 | 16 | >32 | >32 | <=1 | <=0.5 | <=1 | <=0.25 | 00.5 | 4 | 4 | <=2 | >160 | 32 |
| CG1617 | <=1 | <=1 | <=1 | <=1 | <=0.5 | <=1 | <=0.25 | <=0.25 | <=1 | <=1 | <=2 | <=20 | 128 |
| CG1619 | <=1 | <=1 | <=1 | <=1 | <=0.5 | <=1 | <=0.25 | <=0.25 | <=1 | <=1 | <=2 | <=20 | 32 |
| CG1620 | 32 | >32 | >32 | <=1 | <=0.5 | <=1 | 2 | 1 | >8 | >8 | 16 | <=20 | 32 |
| CG1625 | <=1 | <=1 | <=1 | <=1 | <=0.5 | <=1 | <=0.25 | <=0.25 | <=1 | <=1 | <=2 | <=20 | 32 |
| CG1627 | <=1 | <=1 | <=1 | <=1 | <=0.5 | <=1 | <=0.25 | <=0.25 | <=1 | <=1 | <=2 | <=20 | <=16 |
| CG1628 | <=1 | <=1 | <=1 | <=1 | <=0.5 | <=1 | <=0.25 | <=0.25 | <=1 | <=1 | <=2 | <=20 | 32 |
| CG1633 | <=1 | <=1 | 2 | <=1 | <=0.5 | 1 | <=0.25 | <=0.25 | <=1 | <=1 | <=2 | <=20 | 32 |
| CG1645 | <=1 | <=1 | <=1 | <=1 | <=0.5 | <=1 | <=0.25 | <=0.25 | <=1 | <=1 | <=2 | <=20 | 32 |
| CG1646 | 32 | 32 | >32 | <=1 | >=2 | <=1 | <=0.25 | <=0.25 | <=1 | <=1 | <=2 | <=20 | 64 |
| CG1647 | <=1 | <=1 | <=1 | <=1 | <=0.5 | <=1 | <=0.25 | <=0.25 | <=1 | <=1 | <=2 | <=20 | 128 |
| CG1648 | <=1 | <=1 | <=1 | <=1 | <=0.5 | <=1 | <=0.25 | <=0.25 | <=1 | <=1 | <=2 | <=20 | 64 |
| CG1649 | <=1 | <=1 | <=1 | <=1 | <=0.5 | <=1 | <=0.25 | <=0.25 | <=1 | <=1 | <=2 | <=20 | 32 |
| CG1654 | 4 | <=1 | <=1 | <=1 | <=0.5 | 2 | <=0.25 | <=0.25 | <=1 | <=1 | <=2 | <=20 | 32 |
| CG1666 | <=1 | <=1 | <=1 | <=1 | <=0.5 | <=1 | <=0.25 | <=0.25 | <=1 | <=1 | <=2 | <=20 | 32 |
| CG1669 | <=1 | <=1 | <=1 | <=1 | <=0.5 | <=1 | <=0.25 | <=0.25 | <=1 | <=1 | <=2 | <=20 | 64 |
| CG1670 | <=1 | <=1 | <=1 | <=1 | <=0.5 | <=1 | <=0.25 | <=0.25 | <=1 | <=1 | <=2 | <=20 | 64 |
| CG1671 | <=1 | <=1 | <=1 | <=1 | <=0.5 | <=1 | <=0.25 | <=0.25 | <=1 | <=1 | <=2 | <=20 | 64 |
| CG1673 | >32 | >32 | >32 | <=1 | <=0.5 | <=1 | <=0.25 | <=0.25 | <=1 | <=1 | <=2 | <=20 | 32 |
| CG1677 | <=1 | <=1 | <=1 | <=1 | <=0.5 | 2 | <=0.25 | <=0.25 | <=1 | <=1 | <=2 | <=20 | 64 |
| CG1680 | <=1 | <=1 | <=1 | <=1 | <=0.5 | <=1 | <=0.25 | <=0.25 | <=1 | <=1 | <=2 | <=20 | 64 |
| CG1684 | >=16 | >=4 | >=16 | <=2 | <=0.5 | <=1 | <=0.25 | <=0.5 | <=4 | <=4 | <=16 | <=40 | <=32 |
| CG1686 | >32 | >32 | >32 | <=1 | 2 | <=1 | <=0.25 | <=0.25 | <=1 | <=1 | <=2 | <=20 | <=16 |
| CG1690 | <=1 | <=1 | <=1 | <=1 | <=0.5 | <=1 | <=0.25 | 1 | >8 | 8 | <=2 | >160 | 64 |
| CG1694 | <=1 | <=1 | <=1 | <=1 | <=0.5 | 2 | <=0.25 | <=0.25 | <=1 | <=1 | <=2 | <=20 | 32 |
| CG1704 | 16 | >32 | 16 | >32 | <=0.5 | <=1 | >2 | >4 | <=1 | <=1 | <=2 | >160 | 64 |
| CG1705 | >=16 | >=4 | <=4 | >32 | <=0.5 | <=1 | <=0.25 | <=0.5 | <=4 | <=4 | <=16 | <=40 | <=32 |
| CG1708 | <=1 | <=1 | <=1 | <=1 | <=0.5 | <=1 | <=0.25 | <=0.25 | <=1 | <=1 | <=2 | <=20 | 64 |
| CG1710 | <=1 | <=1 | <=1 | <=1 | <=0.5 | <=1 | <=0.25 | <=0.25 | <=1 | <=1 | <=2 | <=20 | 32 |
| CG1712 | <=1 | <=1 | <=1 | <=1 | <=0.5 | <=1 | <=0.25 | <=0.25 | <=1 | <=1 | <=2 | <=20 | 32 |
| CG1713 | <=1 | <=1 | <=1 | <=1 | <=0.5 | <=1 | <=0.25 | <=0.25 | <=1 | <=1 | <=2 | <=20 | 64 |
| CG1720 | <=1 | <=1 | <=1 | <=1 | <=0.5 | <=1 | <=0.25 | <=0.25 | <=1 | <=1 | <=2 | <=20 | 64 |
| CG1722 | <=1 | <=1 | <=1 | <=1 | <=0.5 | <=1 | <=0.25 | <=0.25 | <=1 | <=1 | <=2 | <=20 | 64 |
| CG1727 | <=1 | <=1 | <=1 | <=1 | >=2 | >=4 | <=0.25 | <=0.25 | <=1 | <=1 | <=2 | <=20 | 64 |
| CG1728 | >32 | >32 | >32 | 4 | >=2 | <=1 | <=0.25 | <=0.25 | <=1 | <=1 | <=2 | <=20 | 64 |
| CG1733 | <=1 | 4 | 4 | <=1 | <=0.5 | <=1 | 1 | 1 | <=1 | <=1 | <=2 | <=20 | 64 |
| CG1735 | <=1 | <=1 | <=1 | <=1 | <=0.5 | <=1 | <=0.25 | <=0.25 | <=1 | <=1 | <=2 | <=20 | 64 |
| CG1736 | <=1 | <=1 | <=1 | <=1 | <=0.5 | <=1 | <=0.25 | <=0.25 | <=1 | <=1 | <=2 | <=20 | 32 |
| CG1738 | 4 | >32 | <=1 | >32 | <=0.5 | 2 | <=0.25 | 1 | <=1 | <=1 | <=2 | <=20 | 32 |
| CG1739 | <=1 | <=1 | <=1 | <=1 | <=0.5 | <=1 | <=0.25 | <=0.25 | <=1 | <=1 | <=2 | <=20 | 32 |
| CG1741 | 16 | >32 | >32 | <=1 | <=0.5 | <=1 | >2 | >4 | <=1 | <=1 | <=2 | >160 | 32 |
| CG1743 | <=1 | <=1 | <=1 | <=1 | <=0.5 | <=1 | <=0.25 | <=0.25 | <=1 | <=1 | <=2 | <=20 | 64 |
| CG1746 | >32 | >32 | >32 | >=16 | >=2 | <=1 | >2 | >4 | >8 | >8 | <=2 | >160 | 64 |
| CG1750 | 16 | >32 | >32 | <=1 | <=0.5 | <=1 | >2 | >4 | >8 | 8 | <=2 | >160 | 128 |
| CG1751 | <=1 | 8 | <=1 | <=1 | <=0.5 | <=1 | 2 | 1 | <=1 | <=1 | <=2 | >160 | <=16 |
| CG1761 | <=1 | <=1 | <=1 | <=1 | <=0.5 | <=1 | <=0.25 | <=0.25 | <=1 | <=1 | <=2 | <=20 | 32 |
| CG1762 | 4 | >32 | <=1 | 4 | <=0.5 | <=1 | <=0.25 | 1 | <=1 | <=1 | <=2 | <=20 | 32 |
| CG1763 | 2 | >32 | <=1 | 2 | <=0.5 | <=1 | <=0.25 | 1 | <=1 | <=1 | <=2 | <=20 | 32 |
| CG1765 | <=1 | <=1 | <=1 | <=1 | <=0.5 | 2 | <=0.25 | 1 | <=1 | <=1 | <=2 | <=20 | 32 |
| CG1766 | 2 | >32 | <=1 | 32 | <=0.5 | <=1 | <=0.25 | 1 | <=1 | <=1 | <=2 | <=20 | <=16 |
| CG1774 | <=1 | <=1 | <=1 | <=1 | <=0.5 | 2 | <=0.25 | <=0.25 | <=1 | <=1 | <=2 | <=20 | 64 |
| CG1777 | 16 | 16 | 16 | <=1 | <=0.5 | <=1 | <=0.25 | <=0.25 | <=1 | <=1 | <=2 | <=20 | 32 |
| CG1783 | <=1 | 2 | <=1 | <=1 | <=0.5 | 2 | <=0.25 | 00.5 | <=1 | <=1 | <=2 | <=20 | 128 |
| CG1788 | <=1 | <=1 | <=1 | <=1 | <=0.5 | <=1 | <=0.25 | <=0.25 | <=1 | <=1 | <=2 | <=20 | <=16 |
| CG1790 | 16 | >32 | 16 | <=1 | <=0.5 | <=1 | >2 | >4 | >8 | 8 | <=2 | >160 | 64 |
| CG1794 | <=1 | <=1 | <=1 | <=1 | <=0.5 | <=1 | <=0.25 | <=0.25 | <=1 | <=1 | <=2 | <=20 | 64 |
| CG1795 | <=1 | <=1 | <=1 | <=1 | <=0.5 | <=1 | <=0.25 | <=0.25 | <=1 | <=1 | <=2 | <=20 | 32 |
| CG1797 | <=1 | <=1 | <=1 | <=1 | <=0.5 | <=1 | <=0.25 | <=0.25 | <=1 | <=1 | <=2 | <=20 | 64 |
| CG1798 | <=1 | <=1 | <=1 | <=1 | <=0.5 | <=1 | <=0.25 | <=0.25 | <=1 | <=1 | <=2 | <=20 | 64 |
| CG1799 | >32 | >32 | >32 | <=1 | <=0.5 | <=1 | <=0.25 | <=0.25 | <=1 | <=1 | <=2 | <=20 | 64 |
| CG1801 | <=1 | <=1 | <=1 | <=1 | <=0.5 | <=1 | <=0.25 | <=0.25 | <=1 | <=1 | <=2 | <=20 | 32 |
| CG1802 | >32 | >32 | >32 | <=1 | >=2 | 2 | >2 | >4 | <=1 | <=1 | <=2 | >160 | 64 |
| CG1803 | <=1 | <=1 | <=1 | <=1 | <=0.5 | <=1 | <=0.25 | <=0.25 | <=1 | <=1 | <=2 | <=20 | 64 |
| CG1804 | 16 | >32 | 4 | 8 | <=0.5 | <=1 | >2 | >4 | <=1 | <=1 | <=2 | >160 | <=16 |
| CG1806 | <=1 | <=1 | <=1 | <=1 | <=0.5 | <=1 | <=0.25 | <=0.25 | <=1 | <=1 | <=2 | <=20 | 64 |
| CG1807 | <=4 | >=4 | >=16 | >32 | >=2 | >=4 | <=0.25 | <=0.5 | 8 | 8 | <=16 | >=80 | <=32 |
| CG1808 | <=1 | 2 | <=1 | <=1 | <=0.5 | <=1 | <=0.25 | <=0.25 | <=1 | <=1 | <=2 | <=20 | 64 |
| CG1815 | 16 | 16 | 16 | <=1 | <=0.5 | <=1 | <=0.25 | <=0.25 | <=1 | <=1 | <=2 | <=20 | 32 |
| CG1816 | <=1 | <=1 | <=1 | <=1 | <=0.5 | <=1 | <=0.25 | <=0.25 | <=1 | <=1 | <=2 | <=20 | <=16 |
| CG1817 | <=1 | <=1 | <=1 | <=1 | <=0.5 | <=1 | <=0.25 | <=0.25 | <=1 | <=1 | <=2 | <=20 | 32 |
| CG1818 | 16 | >32 | 4 | 32 | <=0.5 | <=1 | 1 | 1 | <=1 | <=1 | <=2 | <=20 | <=16 |
| CG1819 | <=1 | >32 | >32 | >32 | >4 | >8 | <=0.25 | <=0.25 | <=1 | <=1 | <=2 | <=20 | 64 |
| CG1823 | <=1 | <=1 | <=1 | <=1 | <=0.5 | <=1 | >2 | 4 | <=1 | <=1 | <=2 | <=20 | 64 |
| CG1826 | 16 | 16 | 16 | <=1 | <=0.5 | <=1 | <=0.25 | <=0.25 | <=1 | <=1 | <=2 | <=20 | 32 |
| CG1827 | <=1 | <=1 | <=1 | <=1 | <=0.5 | 2 | <=0.25 | <=0.25 | <=1 | <=1 | <=2 | <=20 | 64 |
| CG1828 | >32 | >32 | >32 | 2 | <=0.5 | <=1 | <=0.25 | <=0.25 | <=1 | <=1 | <=2 | <=20 | 64 |
| CG1829 | <=1 | <=1 | <=1 | <=1 | <=0.5 | <=1 | <=0.25 | <=0.25 | <=1 | <=1 | <=2 | <=20 | 64 |
| CG1830 | <=1 | <=1 | <=1 | <=1 | <=0.5 | <=1 | <=0.25 | <=0.25 | <=1 | <=1 | <=2 | <=20 | <=16 |
| CG1831 | <=1 | <=1 | <=1 | <=1 | <=0.5 | <=1 | <=0.25 | <=0.25 | <=1 | <=1 | <=2 | <=20 | 64 |
| CG1832 | <=1 | >32 | 4 | <=.12 | - | 1 | <=0.25 | <=.12 | <=1 | <=1 | <=2 | <=20 | 32 |

Abbreviations: ATM, aztreonam; CRO, ceftriaxone; CAZ, ceftazidime; IPM, imipenem; ETP, ertapenem; CIP, ciprofloxacin; LVX, levofloxacin; GEN, gentamicin; TOB, tobramycin; AMK, amikacin; NIT, nitrofurantoin; SXT, trimethoprim-sulfamethoxazole; FEP, cefepime; -, no available.
